# Direct molecular mimicry enables off-target cardiovascular toxicity by an enhanced affinity TCR designed for cancer immunotherapy

**DOI:** 10.1038/srep18851

**Published:** 2016-01-13

**Authors:** Marine C C Raman, Pierre J Rizkallah, Ruth Simmons, Zoe Donnellan, Joseph Dukes, Giovanna Bossi, Gabrielle S Le Provost, Penio Todorov, Emma Baston, Emma Hickman, Tara Mahon, Namir Hassan, Annelise Vuidepot, Malkit Sami, David K Cole, Bent K. Jakobsen

**Affiliations:** 1 Immunocore Limited, 101 Park Drive, Milton Park, Abingdon, Oxon, OX14 4RX, United Kingdom; 2 Division of Infection and Immunity, Cardiff University School of Medicine, Henry Wellcome building, Heath Park, Cardiff, CF14 4XN, United Kingdom

## Abstract

Natural T-cell responses generally lack the potency to eradicate cancer. Enhanced affinity T-cell receptors (TCRs) provide an ideal approach to target cancer cells, with emerging clinical data showing significant promise. Nevertheless, the risk of off target reactivity remains a key concern, as exemplified in a recent clinical report describing fatal cardiac toxicity, following administration of MAGE-A3 specific TCR-engineered T-cells, mediated through cross-reactivity with an unrelated epitope from the Titin protein presented on cardiac tissue. Here, we investigated the structural mechanism enabling TCR cross-recognition of MAGE-A3 and Titin, and applied the resulting data to rationally design mutants with improved antigen discrimination, providing a proof-of-concept strategy for altering the fine specificity of a TCR towards an intended target antigen. This study represents the first example of direct molecular mimicry leading to clinically relevant fatal toxicity, mediated by a modified enhanced affinity TCR designed for cancer immunotherapy. Furthermore, these data demonstrate that self-antigens that are expressed at high levels on healthy tissue should be treated with extreme caution when designing immuno-therapeutics.

T-cell immunity is initiated by the interaction between the clonally expressed T-cell antigen receptor (TCR) and peptide-major histocompatibility complexes (pMHC) on target cells^1^. Naturally occurring cancer-reactive TCRs are generally of weak affinity (compared to pathogen-reactive TCRs)^2^,^3^, presumably because of the rigors of thymic selection, and are further disadvantaged because of the low pMHC levels sometimes expressed on tumour cells^4^. However, recent advances have enabled the affinity enhancement of TCRs for their cognate pMHC^5^,^6^,^7^,^8^,^9^,^10^, an approach which has led to the development of novel immunotherapeutics, including TCR-engineered T-cells for adoptive therapy^11^,^12^ and soluble bispecific ImmTACs (immune mobilising monoclonal T-cell receptors against cancer), comprising an affinity enhanced TCR fused to an anti-CD3 antibody fragment. While extensive validation of tissue expression patterns allows for the selection of more disease-specific, and therefore safer, targets TCR off-target cross reactivity is less well-defined and more challenging to assess.

The MAGE-A3 antigen has been a leading target for immunotherapeutic approaches to cancer because of its frequent expression in multiple tumour types and restricted expression in normal tissues^13^,^14^. A wild type TCR recognising the HLA-A*01-restricted MAGE-A3 peptide EVDPIGHLY_168–176_ was isolated and affinity enhanced for use in a T-cell adoptive therapy setting. Extensive preclinical testing of this TCR (termed a3a) gave no concerns regarding recognition of off-target antigens. However, administration of T-cells expressing the a3a TCR resulted in fatal cardiac toxicity in two patients^12^. It was subsequently found, using an amino acid scanning approach, that the a3a TCR unexpectedly recognised an unrelated HLA-A*01-restricted peptide derived from the muscle protein Titin (ESDPIVAQY_394–403_), and that presentation of this peptide on cardiac tissues *in vivo* was the most likely cause of the observed toxicity^15^. While these findings demonstrate the potential of TCR-based therapies to eradicate tumours, they highlighted the pressing need to better understand the mechanisms controlling T-cell cross-reactivity^16^,^17^,^18^,^19^.

A soluble higher affinity variant of the same HLA-A*01-restricted MAGE-A3 wild type TCR from which a3a was derived was engineered for use as an ImmTAC reagent. This TCR (termed MAG-IC3) also showed cross reactivity to the Titin peptide (data shown herein). We therefore sought to investigate the structural basis of the interaction between MAG-IC3 and MAGE-A3/Titin epitopes (termed A1-MAGE-A3 and A1-Titin respectively). Based on these structural data, a rational design approach was followed to introduce additional mutations into the MAG-IC3 sequence in order to alter the fine specificity of the TCR, and thus minimise recognition of A1-Titin whilst maintaining high affinity binding to A1-MAGE-A3. These results demonstrate the structural basis for cross-reactivity of enhanced affinity A1-MAGE-A3 specific TCRs with A1-Titin, and provide a proof-of-concept strategy for altering the fine specificity of a TCR towards an intended target antigen.

## Results

### MAG-IC3 ImmTACs re-direct T-cells to target both A1-MAGE-A3 and A1-Titin presenting cells

**Figure 1:**
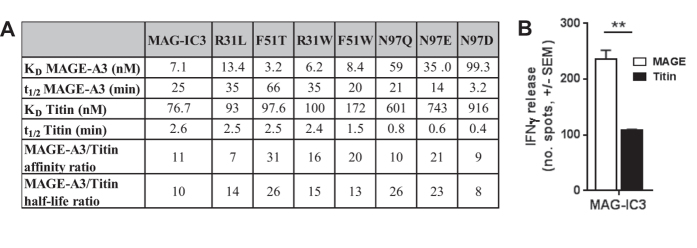
MAG-IC3 TCR targets A1-MAGE-A3 and A1-Titin. (**A**) Single cycle kinetic analysis of MAG-IC3 mutants binding to A1-MAGE-A3 and A1-Titin at 25 °C. For these analyses, approximately 150 RUs of biotinylated pMHC was immobilised onto a CM5 amine chip. The TCR was then injected over the surface using a kinetic injection series. An irrelevant biotinylated pMHC was immobilised onto flow cell 2 as a reference. (**B**) Effector T cells were assessed for reactivity with MAG-IC3 in the presence of HLA-A1-transduced T2 cells pulsed with 2.5 μM peptide (MAGE-A3 or Titin). Reactivity was measured by IFNγ ELIspot assay as described in Materials and Methods. Unpulsed HLA-A*0101 -transduced T2 cells were used as control targets to calculate background activation, which was deducted from the responses to the peptide pulsed targets (data not shown).

To confirm that the soluble high affinity MAG-IC3 TCR was able to recognise both A1-MAGE-A3 and A1-Titin epitopes, binding was investigated by surface plasmon resonance (SPR) and the corresponding ImmTAC reagents assessed for T-cell redirection in the presence of HLA-A*01 transduced T2 cells pulsed with either MAGE-A3 or Titin peptides. The data indicated that the MAG-IC3 TCR bound to both epitopes, but with a slightly weaker affinity and faster half-life recorded for A1-Titin (7.1 nM, and 25 mins compared to 76.7 nM and 2.6 mins) (Fig. 1A, Supplementary Figure 1). ImmTAC reagents made with MAG-IC3 led to potent T-cell activation against both MAGE-A3 and Titin pulsed cells as determined by IFNy release (Fig. 1B). However, in line with the weaker binding affinity and faster half-life, a smaller response was observed against A1-Titin targets (Fig. 1B). Unpulsed HLA-A*0101 -transduced T2 cells were used as control targets to calculate background activation, which was deducted from the responses to the peptide pulsed targets (data not shown).

### The MAG-IC3 TCR uses a conserved binding orientation to engage A1-MAGE-A3 and A1-Titin

**Figure 2:**
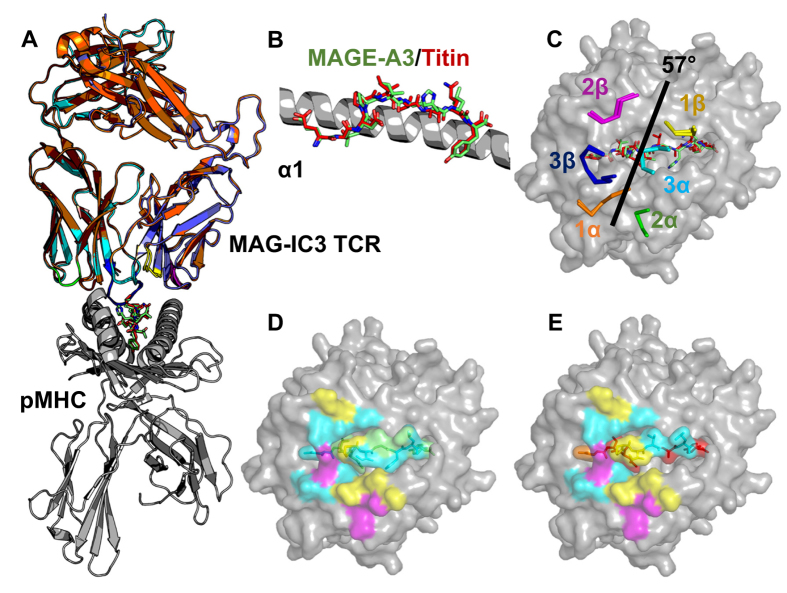
Comparison of MAG-IC3 binding to A1-MAGE-A3 and A1-Titin. (**A**) Superposition of the MAG-IC3 TCR (orange and sand, or cyan and blue cartoon) in complex with HLA-A*0101 (grey cartoon) and the MAGE-A3 (green sticks) and Titin (red sticks) peptides (**B**) Side view superposition of the MAGE-A3 (green sticks) and Titin (red sticks) peptide conformations. (**C**) Aligning of the TCR CDR loops (multi-coloured lines) in complex with the MAGE-A3 (green sticks) and Titin (red sticks) peptides. (**D**) pMHC residues contacted by the TCR in the MAG-IC3-A1-MAGE-A3 complex showing no contacts (grey), 1–2 contacts (cyan), 3–4 contacts (yellow), 7–10 contacts (green), 11–14 contacts (magenta). (**E**) pMHC residues contacted by the TCR in the MAG-IC3-A1-Titin complex showing no contacts (grey), 1–2 contacts (cyan), 3–4 contacts (yellow), 5–6 contacts (orange), 11–14 contacts (magenta), >15 contacts (red).

The A1-MAGE-A3 (EVDPIGHLY) and A1-Titin (ESDPIVAQY) peptides differ in sequence at 4 of 9 positions including positions in the centre of the peptide, which typically dominate TCR contacts^20^. Given that TCR recognition is known to be highly sensitive to differences in peptide sequence^21^,^22^,^23^,^24^, it was unclear how the MAG-IC3 TCR could recognise both peptides. We solved the structure of the MAG-IC3 TCR in complex with both the A1-MAGE-A3 and A1-Titin peptides at atomic resolution. Both structures were solved in space group C 1 2 1 at resolutions between 2.6 and 2.4 Å, with crystallographic R_work_/R_free_ ratios within accepted limits as shown in the theoretically expected distribution^25^ (Supplementary Table 1). The electron density around both peptides was clear and well defined (Supplementary Figure 2). As we have previously reported with other engineered high affinity TCRs^5^,^6^,^26^, MAG-IC3 bound canonically to both ligands, with the TCR α-chain positioned over the MHCI α1-helices and the TCR β-chain over the MHCI α2-helices, enabling the TCR complementarity determining region (CDR) loops to sample the bound peptide^27^,^28^ (Fig. 2A). Despite the differences in peptide sequence between the two ligands, the conformation of the Cα backbone and the orientation of the peptide side chains were very similar (root mean square deviation, or rmsd, based on Cα atoms at 0.285 Å), providing a structural explanation for the cross-reactivity of the A1-MAGE-A3 specific TCR for the A1-Titin self-antigen (Fig. 2B). The similar peptide conformations probably mediated the near identical overall conformation utilised by MAG-IC3 to engage both ligands, evidenced by an rmsd between the two complexes of 0.418Å, based on Cα atoms in the whole model. Further, the positions of the CDR loops and the TCR crossing angle (57 °C) were identical for both complexes (Fig. 2C) and the buried surface area and surface complementarity values were well matched (Table 1). Overall, these data demonstrate that MAG-IC3 utilised a very similar binding mode to engage both A1-MAGE-A3 and A1-Titin, providing a structural explanation for the observed cross-reactivity *via* molecular mimicry.

### An altered contact network between MAG-IC3:MAGE A3 and Titin peptides provide a structural basis for engineering out A1-Titin cross-reactivity

**Figure 3:**
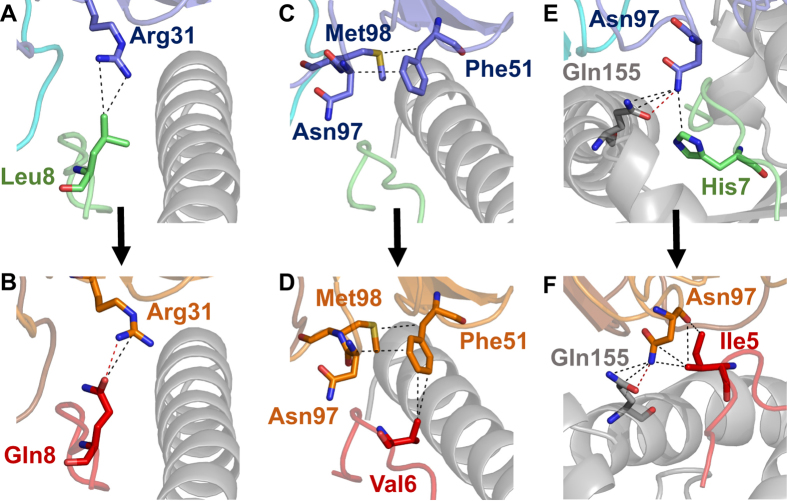
Differences at the contact interface between MAG-IC3 binding to A1-MAGE-A3 versus A1-Titin. (**A**) TCR β-chain residue Arg31 (blue sticks) interaction with MAGE-A3 peptide residue Leu8 (green sticks). (**B**) TCR β-chain residue Arg31 (orange sticks) interaction with Titin peptide residue Gln8 (red sticks). (**C**) TCR β-chain residue Phe51 (blue sticks) position above A1-MAGE-A3 (green and grey cartoon). (**D**) TCR β-chain residue Phe51 (orange sticks) interaction with Titin peptide residue Val6 (red sticks). (**E**) TCR β-chain residue Asn97 (blue sticks) interaction with MAGE-A3 peptide residue His7 (green sticks) and HLA-A*0101 residue Gln155 (grey sticks). (**F**) TCR β-chain residue Asn97 (orange sticks) interaction with Titin peptide residue Ile5 (red sticks) and HLA-A*0101 residue Gln155 (grey sticks). Van der Waals interactions shown as black dotted lines and hydrogen bonds shown as red dotted lines.

We next investigated the fine differences at the interface between MAG-IC3 and the MAGE-A3 peptide and MAG-IC3 and the Titin peptide to explore the possibility of reducing the binding affinity to A1-Titin, whilst retaining recognition of A1-MAGE-A3. Altering the fine specificity of an engineered high affinity TCR using structural data could be an important step in the development of these reagents for therapy. Although MAG-IC3 used a very similar binding mode to engage both pMHCs, there were differences at the TCR-peptide interface focussed around the residues that differed between the peptides (Fig. 2D,E). This analysis revealed 3 TCR residues in which mutations were more likely to affect binding to A1-Titin than A1-MAGE-A3 (Fig. 3). First, TCR residue Arg31β made 1 hydrogen bond (HB) and 1 Van der Waals (VdW) contact with Titin peptide residue Gln8. This HB was not present between TCR residue Arg31β and Leu8 in the MAGE-A3 peptide (only VdW contacts were present) (Fig. 3A,B). Second, TCR residue Phe51β made 2 VdW contacts with Val6 in the Titin peptide, but no contacts with MAGE-A3 (Fig. 3C,D). Third, TCR residue Asn97β made a network of VdW contacts with Titin peptide residue Ile5, compared to a single weak VdW contact with MAGE-A3 residue His7 (Fig. 3E,F). These differences provided potential targets for fine tuning the MAG-IC3 TCR to reduce cross-reactivity with the A1-Titin self-antigen whilst maintaining reactivity towards the tumour target, A1-MAGE-A3.

### Structurally guided TCR mutations have a greater detrimental effect on binding affinity for A1-Titin compared to A1-MAGE-A3

The 3 TCR residue candidates identified by structural analysis, Arg31β, Phe51β and Asn97β, were targeted for mutations that would decrease binding to A1-Titin whilst having no or less effect on the interaction with A1-MAGE-A3. MAG-IC3 bound to A1-Titin with around 10X weaker affinity and 10X shorter half-life compared to A1-MAGE-A3 (Fig. 1A), these binding affinity and half-life ratios between the two ligands served as a baseline for subsequent experiments. It was expected that a larger ratio between the two ligands should enable enhanced TCR discrimination and reduce ImmTAC driven T-cell activation in the presence of A1-Titin presenting cells. In all, thirteen MAG-IC3 mutants were screened by SPR using single injections over A1-MAGE-A3 and A1-Titin immobilised onto a BIAcore CM5 chip (data not shown). This screen identified 7 promising mutations that decreased the binding affinity with A1-Titin, which were subjected to more detailed biophysical analysis (Fig. 1A, Supplementary Figure 1). TCR residue Arg31β, which formed a HB with A1-Titin but not with A1-MAGE-A3, was mutated to Leu and Trp in order to abrogate this interaction, whilst maintaining, or enhancing, hydrophobic interactions with Leu8 in the MAGE-A3 peptide. SPR analysis demonstrated that the R31L mutation decreased the binding affinity (1.9 fold) and half-life (1.4 fold) to A1-MAGE-A3 by a greater extent than those to A1-Titin (1.4 fold and 1.0 fold, respectively). The R31W mutation slightly increased binding affinity (1.1 fold) to A1-MAGE-A3, whilst reducing binding affinity (by 2.2 fold) to A1-Titin, although the reduction in half-life was similar. Thus, neither of the mutations of Arg31β were subjected to further analysis. TCR residue Phe51β, that formed VdW contacts with Val6 in the Titin peptide, but no contacts with A1-MAGE-A3, was mutated to Trp and Thr. We speculated that the large side chain of Trp could increase steric hindrance with the Val6 in the Titin peptide, whilst generating new interactions with Gly6 in the MAGE-A3 peptide. The F51T mutation was intended to abrogate binding between MAG-IC3 and A1-Titin because of the smaller Thr side chain. SPR analysis demonstrated that F51T increased the binding affinity and half-life to A1-MAGE-A3, whilst maintaining the binding affinity with A1-Titin. F51W conserved the binding affinity and half-life to A1-MAGE-A3 whilst decreasing the binding affinity with A1-Titin. The F51T mutation was the most promising mutant because it enhanced the relative ratios compared with MAG-IC3, thus increasing the binding affinity window between MAGE-A3 and Titin by 2.8 fold and the half-life window by 2.6 fold. Finally, TCR residue Asn97β was mutated to Asp, Glu and Gln to increase steric hindrance with Ile5 in the Titin peptide, whilst the longer negatively charged side chains of Asp, Glu and Gln could potentially enable new electrostatic interactions with the positively charged His7 in the MAGE-A3 peptide (Ala7 in the Titin peptide). N97D reduced the binding affinity and half-life with both A1-MAGE-A3 and A1-Titin by around 10 fold. Although both the N97Q and the N97E mutations reduced the binding affinity and half-lives to both MAGE-A3 and Titin, the relative ratios remained the same or were enhanced, increasing binding affinity window between MAGE-A3 and Titin by 1 fold or 2 fold and half-life window by 2.6 fold or 2.3 fold for N97Q and N97E respectively.

### MAG-IC3 mutations enable improved selectivity of ImmTAC targeting using peptide pulsed cell lines

**Figure 4:**
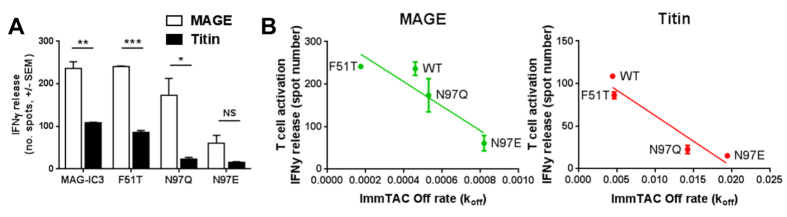
MAG-IC3 TCR mutants enhance specificity towards A1-MAGE-A3 and away from the self-ligand A1-Titin. (**A**) HLA-A*0101-transduced T2 cells which were pulsed with 2.5 μM peptide (MAGE-A3 or Titin) were assessed for reactivity in the presence of MAG-IC3 ImmTAC and the mutant TCR ImmTACs (1 nM). Reactivity was measured by IFNγ ELIspot assay as described in Materials and Methods. Unpulsed HLA-A*0101 -transduced T2 cells were used as control targets to calculate background activation, which was deducted from the responses to the peptide pulsed targets (data not shown). (**B**) Correlation of MAG-IC3, off-rate and T-cell activation for A1-MAGE-A3 and A1-Titin.

To determine whether the mutated reagents could improve discrimination between A1-MAGE-A3 and A1-Titin, we generated ImmTACs using the MAG-IC3 mutants, F51T, N97Q and N97E, and performed a cellular activation assay by pulsing A1-T2 cells with each peptide. T-cell activation was measured as interferon-γ (IFNγ) release in an ELISpot assay. The MAG-IC3 ImmTAC enabled T-cell targeting of A1-MAGE-A3 with around double the IFNγ release of A1-Titin (Fig. 4A). However, the response to A1-Titin was still relatively high. F51T generated only a slight decrease in A1-Titin mediated T-cell activation compared to MAG-IC3, whereas MAGE-A3 mediated T-cell activation was the same for both F51T and MAG-IC3, despite the larger difference in binding affinity and half-life between A1-MAGE-A3 and A1-Titin for the F51T mutant compared to MAG-IC3 (Fig. 4A, Figure 1). N97E reduced A1-Titin mediated T-cell reactivity to near background levels, however, A1-MAGE-A3 mediated T-cell activation by N97E was also reduced by 3.6 fold, reflecting the weaker binding affinity and shorter half-life of the N97E-A1-MAGE-A3 interaction. The N97Q mutation showed the best reduction in A1-Titin mediated T-cell activation (5 fold) whilst maintaining activity mediated by A1-MAGE-A3 (only a 1.2 fold reduction) (Fig. 4A). The N97Q mutation reduced the half-life with A1-Titin from 2.6 to 0.8 minutes compared to 25 to 21 minutes for A1-MAGE-A3 (Fig. 1A). T-cell activation by MAG-IC3 and mutants appeared to be closely related to the off-rate of the molecule (Fig. 4B). In this study, the TCR half-life was a better predictor of activation than the affinity. We postulate that the fast half-life of the N97Q-A1-Titin interaction could be the limiting factor determining whether the ImmTAC reagent can bind to the surface of target cells for long enough to enable re-directed T-cell activation.

### A1-Titin is naturally presented at far higher levels than A1-MAGE-A3

**Figure 5:**
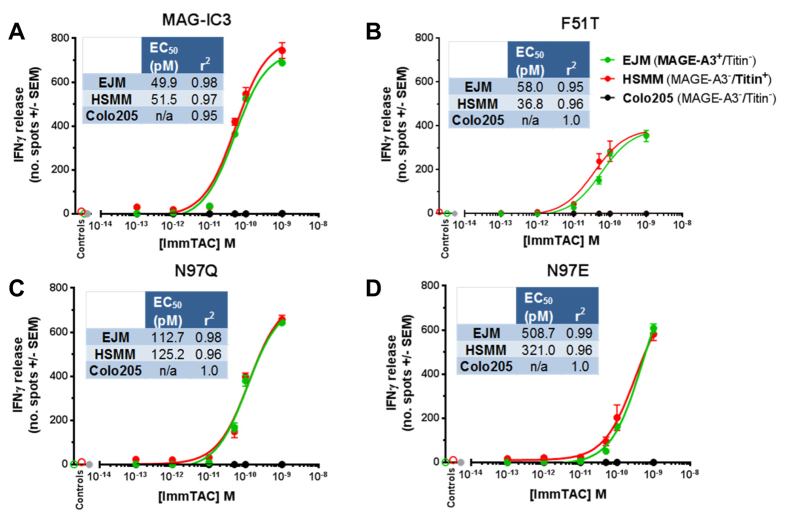
ImmTACs redirect healthy donor PBMCs against HLA-A1+ targets expressing MAGE-A3 and A1-Titin. Cultured human cells were assessed for reactivity with MAG-IC3 ImmTAC and the mutant TCR ImmTACs by ELIspot assays as described in Materials and Methods. The HLA-A1 +/MAGE-A3 +/Titin- multiple myeloma cell line EJM (green line) and HLA-A1 +/MAGE-A3-/Titin + normal Human Skeletal Muscle Myoblast cells (HSMM) (red line) were used as target cells. Expression of HLA-A1 was confirmed using antibody staining (data not shown). HLA-A1 +/MAGE-A3-/Titin- colorectal cell line Colo205 (black line) and PBMCs +1 nM ImmTAC (grey circles) were used as negative controls. Results were analysed including calculations of EC50 and r2 values in GraphPad Prism.

**Figure 6:**
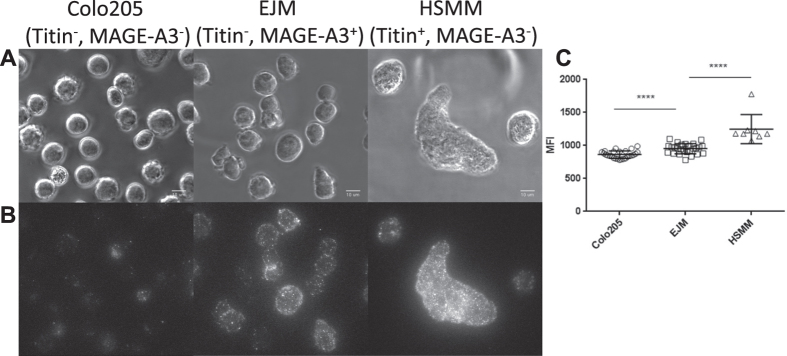
MAGE-A3 a3b3 TCR biotinylated stains strongly enlarged HSMM cells and to a lower extent EJM cells. Colo205 (Titin^−^, MAGE-A3^−^), EJM (Titin^−^, MAGE-A3^+^), HSMM (Titin^+^, MAGE-A3^−^) were stained with MAGE-A3 a3b3 biotinylated TCR. Expression of HLA-A1 was confirmed using antibody staining (data not shown). Phase-contrast and PE-dependent fluorescence images were acquired at room temperature using a 200M/Universal Imaging system with a 63 × objective (Carl Zeiss Inc.). Z-stack fluorescent images were taken (27 individual planes, 0.7 μm apart) to cover the entire 3D extension of the cell. (**A**) Fluorescence images are three-dimensional reconstructions of all the z-stacks acquired as a single plane. (**B**) The phase contrast images corresponding to the fluorescent images are also represented to clearly identify the cells. The brightness/contrast of individual phase contrast and fluorescence images was adjusted to optimise epitope visualisation. The scale bar represents 10 μm. (**C**) Quantification of cells MFI is reported. Statistical significance of data was assessed by un-paired t test (****P < 0.0001).

Although the mutated MAG-IC3 ImmTACs were able to better discriminate between A1-MAGE-A3 and A1-Titin pulsed T2 cells at equivalent concentrations of peptide (Fig. 4A), when these reagents were tested against HLA-A1 positive human cell lines naturally expressing either MAGE-A3 or Titin proteins, they unexpectedly showed little difference in their abilities to trigger a T-cell response (Fig. 5). To investigate whether differences in presentation levels could explain this observation, A1-MAGE-A3 and A1-Titin epitopes were visualised and quantified on target cells using a high-affinity biotinylated mTCR (Fig. 6). These data revealed that A1-Titin was presented at significantly higher levels on Titin positive human skeletal muscle myoblasts (HSMM) (mean fluorescence intensity of 1451), compared to A1-MAGE-A3 on the myeloma cell lines (mean fluorescence intensity of 812) (Fig. 6C). This distinction explains the ability of the mutated MAG-IC3 TCR reagents to effectively target the naturally expressed A1-Titin peptide in spite of their lower affinity and shorter half-life for this antigen.

## Discussion

The affinity of naturally selected TCR for their pMHC target (K_D_ = 0.1–300 μM)^29^, is relatively weak compared to antibodies (K_D_ = nM – pM), despite having a very similar antigen binding domain. The weak affinity of natural TCRs, particularly cancer specific TCRs, severely limits their utility as soluble reagents for targeting diseases. To overcome this limitation, recent advances have enabled the development of enhanced affinity TCRs by altering the sequence of their CDR loops^5^,^6^,^7^,^8^,^9^,^10^. Although these reagents have considerable promise for cancer therapy, modifying the specificity of a TCR outside the rigours of thymic selection does not come without risks considering the cross-reactive potential of TCRs^16^,^17^,^18^,^19^. A clinically relevant example of the dangers of this approach was recently reported in which unexpected cross-reactivity of an enhanced affinity TCR targeting HLA-A*01-restricted epitope from MAGE-A3 (A1-MAGE-A3) resulted in cardiovascular toxicity through recognition of an unrelated HLA-A*01 peptide, A1-Titin^12^,^15^. Here, a structural approach was used to investigate the molecular basis for the observed T-cell cross-reactivity and to determine whether the enhanced affinity reagent could be ‘tuned’ at the atomic level to enable fine discrimination between the cancer target and self-antigen.

Previous structural studies have demonstrated that TCRs generally use a fixed conformation to engage multiple pMHC epitopes^19^,^30^,^31^,^32^,^33^,^34^, with one exception^35^. This first structural evidence of TCR cross-reactivity with a ligand known to have a clinical impact demonstrated that the A1-Titin epitope was a close structural mimic of A1-MAGE-A3, despite differing in sequence at 4 of 9 residues, including at positions in the centre of the peptide that are usually directly involved in contacting the TCR^27^. These observations support the notion that molecular mimicry could be an important pathway enabling T-cell cross-reactivity in a number of diseases, including autoimmune diseases^36^. The similar overall structures of A1-Titin and A1-MAGE-A3 enabled the MAG-IC3 TCR to utilise a virtually identical binding mode to engage both ligands. Despite this similarity, there were still key differences at the interface that facilitated the selection of TCR mutations that could disrupt the MAG-IC3-A1-Titin interaction, whilst having less effect on recognition of A1-MAGE-A3. Three of these mutations were tested as ImmTACs and demonstrated enhanced selectivity for A1-MAGE-A3 in assays using cells pulsed with equivalent concentrations of peptide. The N97Q mutation was particularly effective, despite reducing the binding affinity for both A1-MAGE-A3 and A1-Titin by a similar degree. However, the half-life of the N97Q-A1-Titin interaction was severely shortened (3.2 fold reduction) compared to N97Q-A1-MAGE-A3 (1.2 fold reduction). Similarly, targeting of Titin pulsed cells by N97E (that had a half-life of 36 seconds) was substantially reduced. This distinction could represent an important limit in terms of half-life of these reagents on the surface of a target cell to enable T-cell re-direction. However, when these mutated reagents were tested on A1-MAGE-A3 (cancer) and A1-Titin (normal) positive cells we observed little difference in their abilities to trigger a T-cell response. The ability of the N97E and N97Q mutant TCR ImmTACs to enable T-cell re-direction towards cells naturally expressing A1-Titin was surprising considering the relatively weak binding affinity and short half-life of the reagents. This difference was probably due to the very high levels of Titin self-antigen naturally expressed on the surface of the Human Skeletal Muscle Myoblast cells, enabling even a less potent reagent to effectively target the self-antigen.

In summary, these data demonstrate that a molecular mimicry-like mechanism mediates cross-reactivity enabling an A1-MAGE-A3 specific TCR to target cardiovascular tissue. We also demonstrate a novel structural approach for altering the fine specificity of an enhanced affinity TCR to enable improved antigen discrimination. We have previously demonstrated that enhanced affinity modified TCRs use a native-like binding mode to engage pMHC^5^,^6^,^21^,^22^,^26^. Thus, these results are likely to be relevant to help explain the mechanism of natural TCR cross-reactivity, and demonstrate the possibility of manipulating T-cell antigen specificity using structural information from atomic resolution analysis of the TCR-pMHC interface. Although this approach was successful as a proof-of-concept, the extremely high natural levels of the Titin self-antigen altered the activation threshold required to target this epitope and reduced the potential therapeutic window. A T-cell response was generated towards A1-Titin presenting cells despite drastically reducing the affinity and half-life of the MAG-IC3-A1-Titin interaction. These data demonstrate the extreme sensitivity of the ImmTAC platform and highlight the dangers of unpredictable cross-reactivity, particularly towards self-antigens that are highly expressed in healthy tissues. Predicting and testing for this type cross-reactivity is extremely challenging. In this system, great effort was taken to identify self-reactivity before this therapeutic was administered^12^. Unfortunately in this instance, the cardiovascular toxicity was only observed in patients. Ultimately, our understanding of the molecular basis of TCR cross-reactivity is still in its infancy. Once we understand this process in greater detail, it may become easier to predict this type of unwanted cross-reactivity more accurately.

## Methods

### MAGE A3 TCR engineering

To obtain TCRs with enhanced affinity for A1-MAGE-A3, the wild-type MAGE-A3 TCR was subjected to phage display, as previously described^7^. A panel of high affinity TCRs was obtained with mutations in the α and/or the β chain (data not shown). The MAG-IC3 TCR was selected from this panel because it had the optimal affinity for the A1-MAGE-A3 peptide. Subsequent MAG-IC3 mutants described in this paper were generated by site-directed mutagenesis using MAG-IC3 as a template.

### Protein expression and purification

The MAG-IC3 TCR and mutants, β2m and the HLA-A*0101 chains were cloned into the pGMT7 vector and expressed in the BL21 (DE3) Rosetta pLysS strain as described previously^2^,^37^. The HLA-A*0101 heavy chain was expressed with (for BIAcore experiments), or without (for crystallisation screens) a biotinylation tag, for later refolding with the MAGE-A3 or Titin peptides. The MAG-IC3 TCR was refolded and purified as previously described^38^. For a 250 ml ImmTAC refold, 6.5 mg α chain was mixed with 16 mg β chain. The refolds were extensively dialysed against 20 mM Tris pH8 at 8 °C and purified by Poros50HQ^TM^ 10/100, Poros50HS^TM^ 10/100 (Life Technologies) and Superdex S200HR^TM^ 10/300 (GEH) columns^39^. HLA-A*0101 peptide complexes were refolded and purified as described previously^40^.

### SPR Single cycle kinetic analysis

Purified TCRs were subjected to SPR analysis using a BIAcore3000^TM^. Briefly, A1-MAGE-A3 and A1-Titin were biotinylated as described previously^41^ and were immobilised onto a streptavidin-coupled CM5 sensor chip (approximately 100 RUs of each pMHCI). A control pMHCI monomer was immobilised onto flow cell 2 in all experiments. All measurements were performed at 25 °C in PBS buffer (Sigma) at a flow rate of 50 μl/min. The mutants TCR affinities were determined using single cycle kinetic analysis (sck)^21^,^22^ by analysing the interactions of five concentrations of the TCR ranging from 12.5 nM to 200 nM for A1-MAGE-A3 binding and from 20 nM to 1000 nM for A1-Titin binding. The K_D_ values were calculated assuming Langmuir binding (AB = B x ABmax/(KD + B)), half-lives were calculated from: t1/2 = ln2/ k_off_ and the data were analysed using the kinetic titration algorithm (BIAevaluationTM 3.1)^42^. The data are representative of three independent experiments.

### Cell lines and effector cells

Cell lines used were human skeletal muscle myoblasts (HSMM obtained from Lonza [6F4528]) (HLA-A*0101and Titin positive, MAGE-A3 negative), colorectal adenocarcinoma cells (Colo205 obtained from ATCC [CCL-222]) (HLA-A*0101 positive, MAGE-A3 and Titin negative), multiple myeloma cells (EJM obtained from DSMZ [ACC560]) (HLA-A*0101 and MAGE-A3 positive, Titin negative). Expression of MAGE-A3 and Titin was determined by RT-qPCR analysis (data not shown), and HLA class I status was determined by PCR-SSOP typing (ProImmune). Cells were cultured in suitable conditions recommended by the supplier. T2 cells were obtained from ATCC and transduced in-house with HLA-A*0101 by lentiviral transduction. HLA-A*0101 levels were confirmed by FACS staining. Healthy donor PBMC were used as effector cells which were obtained from Tissue Solutions (AccuCell, Precision Bioservices) or isolated from buffy coats obtained from the National Blood Service.

### Cellular assays

The activity of the ImmTACs was tested by their ability to redirect effector T-cells in the presence of target antigen to produce IFNγ, which was measured by ELIspot assay according to the manufacturer’s instructions (BD BioSciences). Target peptide was either naturally presented by human cells or pulsed on HLA-A*0101-transduced T2 cells. Human cell lines were Assays were set up in R10 (RPMI 1640 containing 10% foetal calf serum, 1% L-glutamine and 1% penicillin/streptomycin (Life Technologies) in a 96 well plate format. Target and effector cells were washed and plated at 50,000 per well each. ELIspot plates were incubated overnight at 37 °C in 5% CO2 and quantified after development using an automated ELIspot reader (ImmunoSpot Series 5 analyser, Cellular Technology Ltd.). Results were analysed, including calculation of EC50 and r2, and statistical t tests were performed using GraphPad Prism. To compare the reactivity of the mutated ImmTACs to MAGE-A3 and Titin, ImmTAC was titrated between 0.0001–1 nM in the presence of human cell lines. Alternatively, 1 nM ImmTAC was used in the presence of HLA-A*0101 -transduced T2 cells and 2.5 μM MAGE-A3 or Titin peptide. Unpulsed HLA-A*0101 -transduced T2 cells were used as control targets to calculate background activation, which was deducted from the responses to the peptide pulsed targets.

### Crystal structure determination

All protein crystals were grown at 18 °C by vapour diffusion via the sitting drop technique. MAG-IC3-A1-MAGE-A3 crystals were grown in 0.2 M ammonium chloride, 0.1 M HEPES pH7, 15% PEG 8000^43^; MAG-IC3-A1-Titin crystals were grown in 0.2 M ammonium chloride, 0.1 M MES pH7, 20% PEG 4000^43^. Crystallisation screens were conducted using an Art-Robbins Phoenix dispensing robot (Alpha Biotech Ltd, UK) and data were collected at 100 K at the Diamond Light Source (DLS), Oxfordshire, UK at a wavelength of 0.98 Å using an ADSC Q315 CCD detector. Reflection intensities were estimated using XDS^44^ via the XIA2 package^45^ and the data were scaled and merged with SCALA and the CCP4 package^46^. Structures were solved with molecular replacement using PHASER^47^. Sequences and model manipulations were conducted with COOT^48^ and the models refined with REFMAC5. Graphical representations were prepared with PYMOL^49^. The reflection data and final model coordinates were deposited with the PDB database (MAG-IC3-A1-MAGE-A3 PDB: 5BRZ, MAG-IC3-A1-TitinPDB: 5BS0).

### Microscopy

Colo205 (Titin^−^, MAGE-A3^−^), EJM (Titin^−^, MAGE-A3^+^), HSMM (Titin^+^, MAGE-A3^−^) were stained with 5 μg/ml MAGE-A3 a3b3 biotinylated TCR. Expression of HLA-A1 was confirmed using antibody staining (data not shown). Phase-contrast and PE-dependent fluorescence images were acquired as previously described^50^ using a 200M/Universal Imaging system with a 63 × objective (Carl Zeiss Inc.). Z-stack fluorescent images were taken (27 individual planes, 0.7 μm apart) to cover the entire 3D extension of the cell. The images represented are a 3D projection of the Z-stacks acquired with MetaMorph 7.7 imaging program. Single cells MFI was determined with MetaMorph algorithm and statistical analysis was performed with GraphPad Prism 6.05. In each experiment epitopes were quantified on more than 18 individual cells.

## Additional Information

**How to cite this article**: Raman, M. C. C. *et al.* Direct molecular mimicry enables off-target cardiovascular toxicity by an enhanced affinity TCR designed for cancer immunotherapy. *Sci. Rep.* **6**, 18851; doi: 10.1038/srep18851 (2016).

## Supplementary Material

Supplementary Information

## Figures and Tables

**Table 1:** Structural summary of MAG-IC3-A1-MAGE-A3 and MAG-IC3-A1-Titin structures.

Structural Features	MAG-IC3-A1-MAGE-A3	MAG-IC3-A1-Titin
Short Polar (≤3.2 Å)	9	13
Long Polar (≤3.4 Å)	8	5
Short vdW (≤3.5 Å)	13	7
Long vdW (≤4 Å)	82	97
Total contacts	112	122
Number of α chain CDR1/CDR2/CDR3contacts (≤4 Å)	10/40/32	6/48/38
Number of β chain CDR1/CDR2/CDR3contacts (≤4 Å)	2/17/11	2/16/12
Peptide contacts	16	26
MHC contacts	96	96
Crossing Angle	57.0°	57.3°
Buried surface area (TCR-MHC) (Å^2^)	1514.8	1603.8
Buried surface area (TCR-peptide) (Å^2^)	538.4	624.0
Buried surface area (TCR-pMHC) (Å^2^)	2053.2	2227.9
Surface complementarity (TCR-MHC)	0.663	0.671
Surface complementarity (TCR- peptide)	0.470	0.608
Surface complementarity (TCR-pMHC)	0.608	0.661
