# Supplementary material for: Direct molecular mimicry enables off-target cardiovascular toxicity by an enhanced affinity TCR designed for cancer immunotherapy
Source: Sci Rep. 2016 Jan 13;6:18851. doi: 10.1038/srep18851 (PMC4725365; doi:10.1038/srep18851)
Supplement: Supplementary Information [file srep18851-s1.pdf]

Supplementary data

Direct molecular mimicry enables off-target cardiovascular toxicity by an enhanced affinity TCR designed for cancer immunotherapy

Marine C C Raman[1,3], Pierre J Rizkallah[2,3], Ruth Simmons[1], Zoe Donnellan[1], Joseph Dukes[1], Giovanna Bossi[1], Gabrielle S Le Provost[1], Penio Todorov[1], Emma Baston[1], Emma Hickman[1], Tara Mahon[1], Namir Hassan[1], Annelise Vuidepot[1], Malkit Sami[1,3], David K Cole[2,3*], Bent K. Jakobsen[1,3*]

[1] Immunocore Limited, 57-59 Milton Park, Abingdon, Oxon, OX14 4RX, United Kingdom.
[2] Division of Infection and Immunity, Cardiff University School of Medicine, Henry Wellcome building, Heath Park, Cardiff, CF14 4XN, United Kingdom.
[3] These authors contributed equally to this work
[*] Correspondence: Dr Bent Jakobsen, Immunocore Limited, 57-59 Milton Park, Abingdon, Oxon, OX14 4RX, United Kingdom. E-mail: bent.jakobsen@immunocore.com, or Dr David Cole, Division of Infection and Immunity, Cardiff University School of Medicine, Henry Wellcome building, Heath Park, Cardiff, CF14 4XN, United Kingdom. E-mail: coledk@cf.ac.uk.

	MAG-IC3-A1-MAGE-A3	MAG-IC3-A1-TITIN
**Data Collection**		
Beamline	Diamond I02	Diamond I02
Wavelength (Å)	0.9795	0.9795
Space Group	C 1 2 1	C 1 2 1
Cell Dimensions		
a, b, c (Å)	173.59, 47.50, 119.25	173.81, 47.48, 119.53
a, b, g (°)	90.0, 109.1, 90.0	90.0, 109.4, 90.0
Wilson B-factor (Å^2)	52.0	41.7
Resolution Range (Å)	82.01-2.62	81.99-2.40
Outer shell (Å)	2.69-2.62	2.46-2.40
Total Reflections	99,682 (7,447)	131,025 (10,347)
Unique Reflections	27,832 (2,005)	36,248 (2,672)
Work / Test Reflections	26.433 / 1,399	34,441 / 1,807
Redundancy	3.6 (3.7)	3.6 (3.9)
Completeness (%)	98.9 (99.7)	99.1 (100)
Mean I/s(I)	12.4 (1.9)	9.2 (1.9)
R_{merge} (%)	8.8 (91.5)	8.8 (75.5)
**Refinement statistics**		
Non-H atoms	6618	6635
R_{work} / R_{free} (%)	19.3 / 26.8	20.3 / 26.2
$B_{Average}$ (Å^2) mc, sc+sol, all*	61.6, 64.6, 63.3	55.0, 58.5, 56.8
Protein / Non-protein	63.2	55.1
rmsd bond lengths (Å)	0.012	0.014
rmsd bond angles (°)	1.597	1.700

* Main chain, side chain + solvent + ligand + ion, all non-H atoms
One crystal was used to solve each structure
Highest resolution shell is shown in parenthesis

Supplementary Table 1

MAGE Residue	MAG-IC3 Residue	Short Polar (≤3.2Å)	Long Polar (≤3.4Å)	vdW < 3.5	vdW <4.0
Glu 1	aAla 98	1			3
Pro 4	aTyr 32				1
Pro 4	aPhe 101				1
Pro 4	bArg 56	1			5
Ile 5	bAla 99				1
His 7	bAsn 97			1	
Leu 8	bArg 31			1	1
**MHC Residue**	**MAG-IC3 Residue**				
Gln 62	αGly 99		1		
Gln 62	αPro 100				1
Arg 65	βGln 55	1			2
Asn 66	βArg 56	1			5
Ala 69	βArg 56			1	1
Val 150	βPhe 96				2
His 151	αArg 52				4
Glu 154	αArg 52	2		4	4
Glu 154	αPro 53			1	2
Gln 154	α Tyr 54				1
Gln 155	αTyr 32			1	1
Gln 155	βAsn 97	1			2
Gln 155	βAla 99				3
Gln 155	βThr 100				1
Arg 157	α Tyr 54		2	2	13
Val 158	αIle 31				2
Val 158	αTyr 32				4
Val 158	αPro 53				1
Val 158	α Tyr 54		1	1	2
Tyr 159	αTyr 32				1
Arg 163	αGly 96	1			2
Arg 163	αGly 97		2	1	7
Arg 163	αAla 98	1			2
Arg 163	αGly 99		2		
Arg 163	αPhe 101				3
Asp 166	αGly 97				2
Asp 166	αAla 98				2

MAG-IC3-A1-MAGE-A3 contact table

Supplementary Table 2

Titin Residue	MAG-IC3 Residue	Short Polar (≤3.2Å)	Long Polar (≤3.4Å)	vdW < 3.5	vdW <4.0
Glu 1	αAla 98	2	1	2	5
Pro 4	αPhe 101				1
Pro 4	βArg 56	1			3
Pro 4	βMet 98				1
Ile 5	αTyr 32				2
Ile 5	βAsn 97				4
Val 6	βPhe 51				2
Gln 8	βArg 31	1			1
MHC Residue	MAG-IC3 Residue				
Gln 62	αGly 99	1			1
Gln 62	αPhe 101				2
Arg 65	βThr 54				1
Arg 65	βGln 55	1			4
Arg 65	βArg 56				1
Asn 66	βArg 56	1			1
Ala 69	βArg 56				1
His 151	αArg 52				6
Glu 154	αArg 52	1	2	3	7
Glu 154	αPro 53				3
Glu 154	αTyr 54				1
Gln 155	αTyr 32				2
Gln 155	βAsn 97	1			2
Gln 155	βAla 99				3
Gln 155	βThr 100				1
Arg 157	α Tyr 54				19
Val 158	αPro 53				2
Val 158	α Tyr 54				3
Val 158	αGly 96				1
Tyr 159	αTyr 32				1
Gly 162	αGly 30				1
Arg 163	αGly 96		1		4
Arg 163	αGly 97	2	1	2	2
Arg 163	αAla 98	1			3
Arg 163	αGly 99	1			2
Arg 163	αPhe 101				1
Asp 166	αGly 97				1
Asp 166	αAla 98				2

MAG-IC3-A1-Titin contact table

Supplementary Table 3

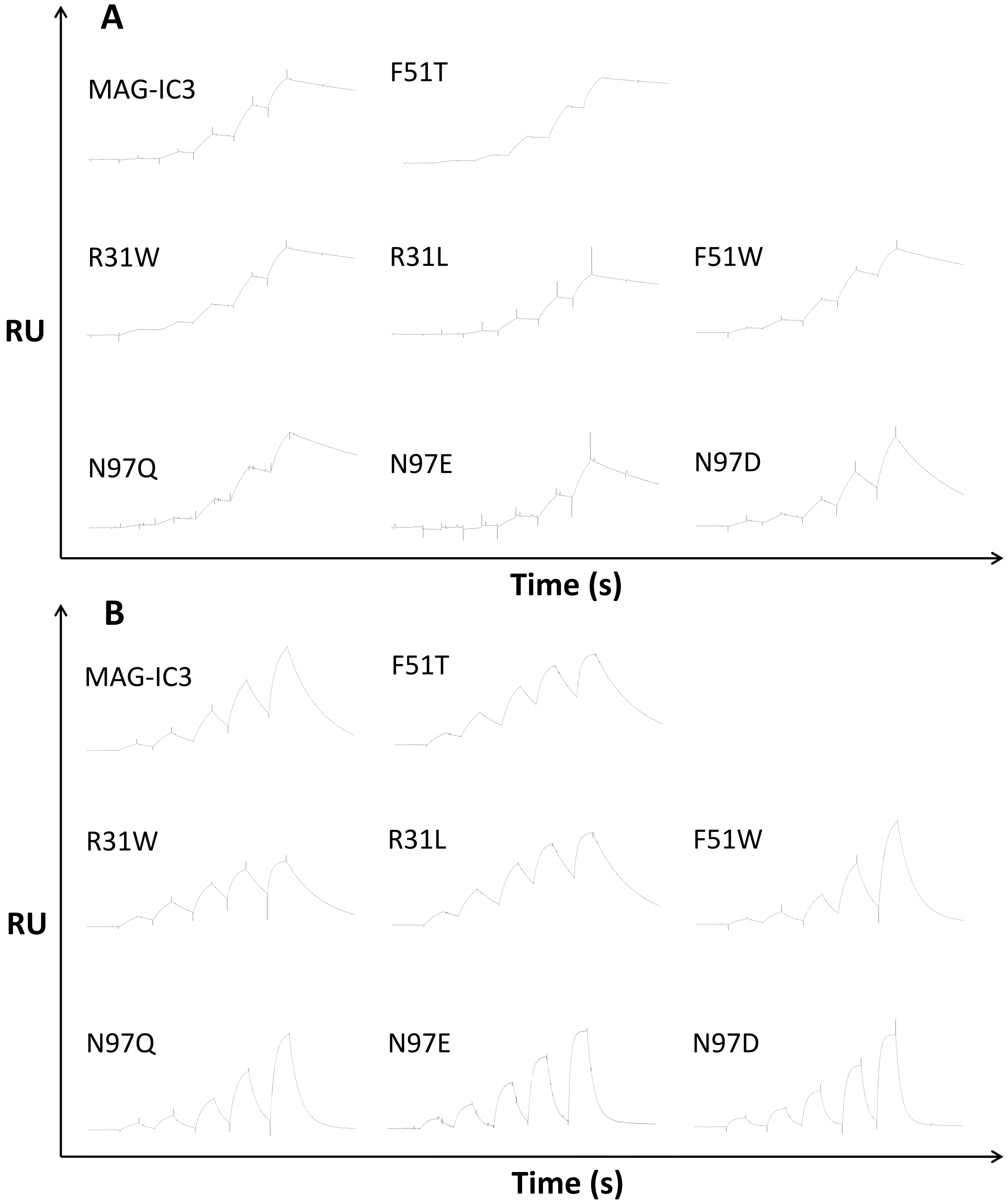

Supplementary Figure 1: Biophysical analysis of MAG-IC3 and mutants with both A1-MAGE-A3 and A1-Titin. Single cycle kinetic analysis of MAG-IC3 and mutants binding to (**A**) A1-MAGE-A3 and (**B**) A1-Titin at 25°C. For these analysis, approximately 150 RUs of biotinylated pMHC was immobilised onto a CM5 amine chip. Each ImmTAC was injected over the surface using a kinetic injection series. 1500 seconds of the dissociation period is shown. An irrelevant biotinylated pMHC was immobilised onto flow cell 2 as a reference.

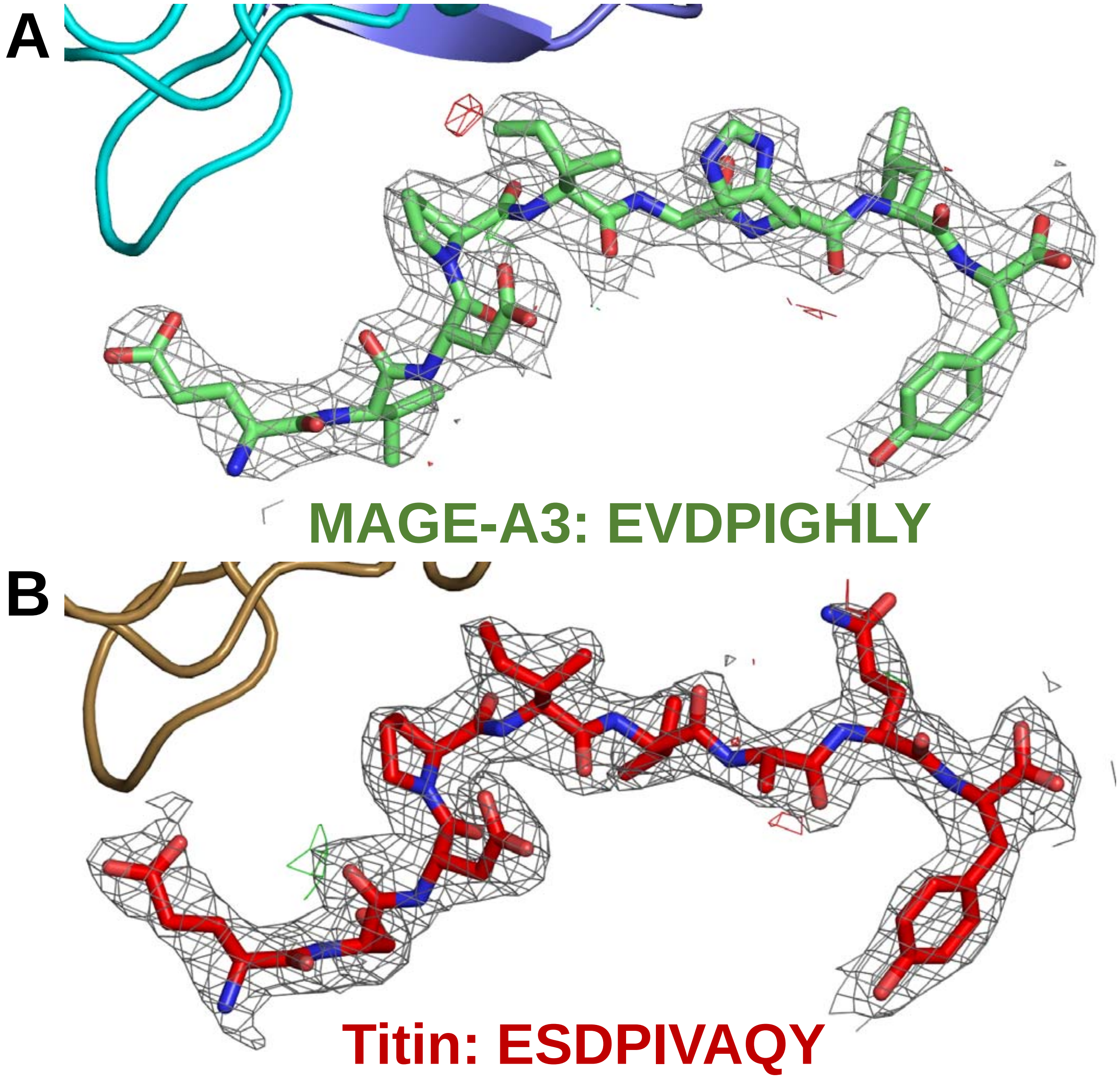

Supplementary Figure 2: Electron density map. 2Fo-Fc observed electron density maps at 2 sigma (shown in grey) for (**A**) A1-MAGE-A3 peptide (green sticks) and (**B**) A1-Titin peptide (red sticks). All maps shown are within 2Å from the atoms to which they relate. Positive density is shown in green and negative density is shown in red.
